# Cannabidiol as a Potential New Type of an Antipsychotic. A Critical Review of the Evidence

**DOI:** 10.3389/fphar.2016.00422

**Published:** 2016-11-08

**Authors:** Cathrin Rohleder, Juliane K. Müller, Bettina Lange, F. M. Leweke

**Affiliations:** Department of Psychiatry and Psychotherapy, Central Institute of Mental Health, Medical Faculty Mannheim, Heidelberg University, MannheimGermany

**Keywords:** schizophrenia, psychosis, animal models, clinical trials, CBD

## Abstract

There is urgent need for the development of mechanistically different and less side-effect prone antipsychotic compounds. The endocannabinoid system has been suggested to represent a potential new target in this indication. While the chronic use of cannabis itself has been considered a risk factor contributing to the development of schizophrenia, triggered by the phytocannabinoid delta-9-tetrahydrocannabinol (Δ^9^-THC), cannabidiol, the second most important phytocannabinoid, appears to have no psychotomimetic potential. Although, results from animal studies are inconsistent to a certain extent and seem to depend on behavioral paradigms, treatment duration and experimental conditions applied, cannabidiol has shown antipsychotic properties in both rodents and rhesus monkeys. After some individual treatment attempts, the first randomized, double-blind controlled clinical trial demonstrated that in acute schizophrenia cannabidiol exerts antipsychotic properties comparable to the antipsychotic drug amisulpride while being accompanied by a superior, placebo-like side effect profile. As the clinical improvement by cannabidiol was significantly associated with elevated anandamide levels, it appears likely that its antipsychotic action is based on mechanisms associated with increased anandamide concentrations. Although, a plethora of mechanisms of action has been suggested, their potential relevance for the antipsychotic effects of cannabidiol still needs to be investigated. The clarification of these mechanisms as well as the establishment of cannabidiol’s antipsychotic efficacy and its hopefully benign side-effect profile remains the subject of a number of previously started clinical trials.

## Introduction

*Cannabis sativa* has been known and used by humans for several 1000 years and the knowledge that it contains an intoxicating principle dates back to 1000 to 1500 B.C. (Adams, 1942). The two major compounds of cannabis – delta-9-tetrahydrocannabinol (Δ^9^-THC) and cannabidiol – have been chemically identified in the 1940th (Adams et al., 1940a,b,c; Adams, 1942; Todd, 1946). Two decades later, remaining uncertainties regarding the exact position of double bonds were eliminated as new imaging techniques like NMR spectroscopy and X-ray structure determination became available (Mechoulam and Shvo, 1963; Gaoni and Mechoulam, 1964; Jones et al., 1977).

Along with its chemical identification, Δ^9^-THC has been identified as the major pro-psychotic compound of *Cannabis sativa* (Adams and Baker, 1940; Adams et al., 1940b; Allentuck and Bowman, 1942; Wollner et al., 1942; Mechoulam et al., 1970). However, the underlying neurobiological principles remained conjectural until it was observed that cannabinoid drugs inhibit adenylate cyclase activity in neuroblastoma cells (Howlett, 1984), and the subsequent discovery of the G-protein coupled type 1 cannabinoid receptor (CB_1_R) (Devane et al., 1988; Matsuda et al., 1990). A few years later, the type 2 cannabinoid receptor (CB_2_R) (Munro et al., 1993) as well as the two major endogenous ligands to cannabinoid receptors – anandamide (Devane et al., 1992) and 2-arachidonoyl-*sn*-glycerol (Mechoulam et al., 1995; Stella et al., 1997) – were discovered. The architecture of the endocannabinoid system (ECS) – including endocannabinoids, cannabinoid receptors as well as synthesizing and degrading enzymes – has been vividly summarized and illustrated in recent reviews by Lutz et al. (2015) and Lu and Mackie (2016).

Studies indicating that cannabis abuse might be a stressor for psychotic relapse and exacerbation of schizophrenic symptoms as well as the observation that Δ^9^-THC induced schizophrenia-like neuropsychological and psychopathological alterations in healthy volunteers, led to the hypothesis that a dysfunctional ECS is involved in the etiology of psychoses (Emrich et al., 1997). In the meantime, several studies reported diverse acute Δ^9^-THC effects in healthy participants and schizophrenic patients, thereby confirming the ECS hypothesis (Leweke et al., 1999, 2000; D’Souza et al., 2004, 2005; Koethe et al., 2006; Bhattacharyya et al., 2009; Fusar-Poli et al., 2009; Koethe et al., 2009; Mason et al., 2009; Sherif et al., 2016). In addition, several epidemiological studies (for review see, Gage et al., 2016) substantiated the view that cannabis use has to be considered an important environmental risk factor for the development of schizophrenia in vulnerable individuals. However, the actual lifetime risk seems to be influenced by the dose (Zammit et al., 2002; Moore et al., 2007) and frequency of cannabis consumption (Di Forti et al., 2009), the potency of consumed cannabis preparations (Di Forti et al., 2009, 2014) and age of onset (Arseneault et al., 2002).

While a dysfunctional ECS seems to contribute to the pathophysiology of schizophrenia, the endocannabinoid anandamide is considered to have protective effects by counteracting neurotransmitter imbalances (Leweke, 2012). Therefore, it has been suggested that modulating the ECS might be a new, promising pharmacological target for schizophrenia.

To date, two main approaches targeting the ECS have been systematically studied in humans: first, trials using CB_1_R antagonists to treat both psychotic and cognitive symptoms of schizophrenia, and, second, trials using the second most important phytocannabinoid cannabidiol (Leweke et al., 2016). In addition, a single clinical case series on dronabinol (Δ^9^-THC) in treatment-refractory severe chronic schizophrenia has been conducted (Schwarcz et al., 2009).

In contrast to Δ^9^-THC, cannabidiol appears to have no psychotomimetic potential, but shows antipsychotic effects in rodents and humans. Thus, this review focuses on (1) preclinical studies investigating cannabidiol as a potential antipsychotic in animal models of aspects of schizophrenia, (2) clinical evidence for its antipsychotic action, and, (3) potential mechanisms of action and their potential relevance for the antipsychotic effects of cannabidiol.

## Methods

We conducted a PubMed search up to and including June 28, 2016, using the search terms cannabidiol AND (antipsychotic OR schizophrenia OR psychosis). Identified references were scanned for clinical trials with schizophrenic patients as well as studies with animal models of aspects of schizophrenia, analyzing the effects of cannabidiol on negative symptoms and cognitive deficits. Studies restricted to analysis of locomotor activity or anxiety were not considered. In addition, we used the search term cannabidiol AND targets to scan for reports on the possible mode of action.

## Antipsychotic Potential Of Cannabidiol: Insights From Preclinical Studies

Schizophrenia is characterized by heterogeneous symptoms that can be grouped into three main symptom categories: (1) positive symptoms (delusions, thought disorder, hallucinations), (2) negative symptoms (anhedonia, blunted affect, social withdrawal) and (3) cognitive impairment (sensory information processing, attention, working memory, executive functions) (Freedman, 2003; Wong and Van Tol, 2003). These main symptoms are often accompanied by more unspecific symptoms like anxiety (Freedman, 2003).

Although psychotic symptoms are human specific to a large extent, animal models are able to provide insight into certain aspects of schizophrenia, including negative symptoms [e.g., inadequate social behavior/social withdrawal, sensorimotor gating deficits as measured by prepulse inhibition (PPI)], cognitive impairments (e.g., working memory deficits) or anxiety. Thus, these animal models for aspects of schizophrenia can be used to investigate the antipsychotic potential of new drugs like cannabidiol on negative and cognitive symptoms. As available antipsychotics do not sufficiently ameliorate negative symptoms and cognitive impairments (Hanson et al., 2010), these studies can make an important contribution.

Schizophrenia is not only characterized by a heterogeneous combination of symptoms but also by a heterogeneous etiology (Cannon and Jones, 1996; Brown, 2011; Kahn et al., 2015). This led to the development of animal models based on different etiological factors of schizophrenia. In the following, the concepts of animal models used to study the effects of cannabidiol are summarized in brief.

As mentioned above, cannabis use is regarded as one important risk factor for the development of schizophrenia. Hence, animal models of early cannabis exposure are used to investigate the long-lasting behavioral consequences of cannabis use and to clarify the underlying cellular mechanisms (Rubino and Parolaro, 2016). However, based on the observation that cannabis preparations or single cannabinoids like Δ^9^-THC induce psychotic-like symptoms in healthy volunteers (for review see Sherif et al., 2016), acute cannabinoid administration is also used to mimic schizophrenia-like symptoms in rodents.

Furthermore, based on the hypothesis that disrupted glutamatergic neurotransmission contributes to the development of schizophrenia (Kim et al., 1980), pharmacological and genetic glutamatergic models have been used in cannabidiol research. Since this dysfunction is characterized by hypofunctional *N*-methyl-D-aspartic acid (NMDA) receptors (for review see Snyder and Gao, 2013), for example, NMDA receptor antagonists like MK-801 are administered to mimic schizophrenic-like symptoms in rodents. The genetic model is based on *neuregulin 1*, a susceptibility gene for schizophrenia (Stefansson et al., 2002). *Neuregulin 1* is involved in neuronal migration, influences myelination and regulates expression of NMDA, γ-aminobuytric acid receptor A (GABA_A_) as well as acetylcholin receptor subunits (for review see Corfas et al., 2004). Heterozygous transmembrane *Neuregulin 1* mutant mice (*Nrg1* TM HET) seem to have fewer functional NMDA receptors (Stefansson et al., 2002), a region-specific alteration of NMDA receptor expression as well as decreased dopamine D_2_ receptor binding in the striatum (Newell et al., 2013). Interestingly, the CB_1_ receptor density is comparable to the density in wild type animals except for a slight increase within the striatum of *Nrg1* TM HET (Newell et al., 2013).

The spontaneously hypertensive rat (SHR) strain has also been suggested as a model for aspects of schizophrenia. These rats show impaired social interaction (Calzavara et al., 2011; Almeida et al., 2014) and reduced PPI (Levin et al., 2011, 2014) as compared to Wistar rats. In addition, antipsychotic drugs reduced abnormalities in contextual fear conditioning (Calzavara et al., 2009), social interaction (Calzavara et al., 2011) as well as PPI (Levin et al., 2011). Nevertheless, other studies observed an increased PPI compared to Sprague Dawely rats (van den Buuse, 2004) or an increased social interaction behavior toward Wistar-Kyoto rats (Hopkins et al., 2009).

### Effects of Cannabidiol on Social Behavior

Social withdrawal is a key negative symptom of schizophrenia. Thus, several studies investigated the effects of cannabidiol on social behavior in different rodent animal models for schizophrenia (**Table 1**).

**Table 1:** Animal studies evaluating the effects of cannabidiol (CBD) on social behavior.

Animal model	Treatment regimen and test procedure	Effective dose [/kg]	Reference
Spontaneously hypertensive rats (SHR)	1, 5, 15, 30, or 60 mg/kg CBD, i.p. injection 30 min prior to social interaction test	–	Almeida et al., 2013
MK-801 (acute, 0.3 or 0.6 mg/kg), male Sprague Dawley rats	1 or 3 mg/kg CBD, i.p. injection 20 min prior to MK-801 administration. Social interaction test started 20 min after the last injection	3, 10 (partially)	Gururajan et al., 2011
MK-801 (acute, 0.3 mg/kg), male Sprague Dawley rats	1 or 3 mg/kg CBD, i.p. injection 20 min prior to MK-801 administration. A modified social interaction test started 20 min after the last injection	3	Gururajan et al., 2012
MK-801 (chronic: 1 mg/kg, 28 days), male C57BL/6J mice	30 or 60 mg/kg CBD, i.p. injection 30 min prior to social interaction test	60	Gomes et al., 2015b
MK-801 (acute, 0.08 mg/kg), male Wistar rats	5, 12, or 30 mg/kg CBD, i.p. injection 30 min prior to MK-801 administration. Social interaction/recognition test started 30 min after the last injection	–	Deiana et al., 2015
Male *Nrg1* TM HET mice	Chronic treatment with 1, 50, or 100 mg/kg CBD over 3 weeks	50 (partially 1 and 100)	Long et al., 2012
Δ^9^-THC (1 mg/kg), male Sprague Dawley rats	5 or 20 mg/kg CBD, i.p. injection 20 min prior to Δ^9^-THC administration. Social interaction test started 20 min after the last injection	20	Malone et al., 2009

Cannabidiol (dosage range: 1–50 mg/kg) itself seems to have no effect on social interaction of untreated Sprague Dawley (Malone et al., 2009; Gururajan et al., 2012), Wistar rats (van Ree et al., 1984; Deiana et al., 2015), C57BL/6JArc mice (Long et al., 2010; Gomes et al., 2015b), and wild type-like littermates of *Nrg1* TM HET mice (Long et al., 2012). However, in Wistar rats 1 mg/kg cannabidiol increased social interaction behavior, whereas higher dosages (5, 15, 30, 60 mg/kg) had no effect (Almeida et al., 2013). In addition, impaired social memory was observed in Wistar rats (Deiana et al., 2015) after acute cannabidiol administration (12 and 30 mg/kg, but not 5 mg/kg).

The majority of studies reported that cannabidiol was able to attenuate or reverse induced altered social behavior. Pretreatment with 20 mg/kg cannabidiol reversed the effects of 1 mg/kg Δ^9^-THC (Malone et al., 2009), while 3 mg/kg cannabidiol inhibited the effects on social investigative behavior of acute MK-801 treatment in a modified social interaction task, increasing it significantly beyond control level (Gururajan et al., 2012). This is in line with the previous finding of the group that cannabidiol (3, 10 mg/kg) partially inhibited MK-801-induced social withdrawal in a classical social interaction paradigm (Gururajan et al., 2011). In mice, pretreatment with 60 mg/kg cannabidiol was found to reverse impaired social interaction induced by chronic MK-801 treatment, while a lower cannabidiol dose (30 mg/kg) attenuated the effects of MK-801 only by trend (Gomes et al., 2015b). As the antipsychotic clozapine also inhibited MK-801 effects on social investigative behavior, it has been suggested that cannabidiol might also be effective in schizophrenia patients showing inadequate social behaviors (Gururajan et al., 2012; Gomes et al., 2015b). However, cannabidiol did not reverse the social recognition impairments induced by an acute low-dose injection of MK-801 in Wistar rats. Furthermore, cannabidiol was not able to elevate the decreased social interaction of SHR rats (Almeida et al., 2013).

Although, *Nrg1* TM HET mutant mice showed similar social behavior compared to their wild type-like littermates (Long et al., 2012), chronic cannabidiol treatment (50 mg/kg, 21 days) increased social interaction as well as specific social behaviors like nosing and anogenital sniffing in mutant mice but not in wild type mice. A higher cannabidiol dose (100 mg/kg) solely increased anogenital-sniffing duration in mutant mice, while lower concentrations (1 mg/kg) led only to increasing nosing frequencies.

Taken together, cannabidiol showed antipsychotic properties in glutamatergic animal models as well as in a model targeting the ECS, whereas it has been ineffective in SHR rats.

### Effects of Cannabidiol on Prepulse Inhibition

Prepulse inhibition of the acoustic startle response is a neuropsychological process during which a weak sensory stimulus – prepulse – attenuates the motor response to a subsequent strong startling stimulus (Rohleder et al., 2016). Since PPI impairments are observed in schizophrenia patients and PPI can be reliably assessed in both animals and humans, it has been used as behavioral measure of aspects of schizophrenia. Unfortunately, results from rodent studies analyzing the effects of cannabidiol on PPI and acoustic startle response in animal models of schizophrenia and untreated control rodents are inconsistent to a certain extent (**Table 2**).

**Table 2:** Animal studies evaluating the effects of cannabidiol (CBD) on prepulse inhibition (PPI).

Animal model	Treatment regimen and test procedure	Effective dose [mg/kg]	Reference
Spontaneously hypertensive rats (SHR)	15, 30, or 60 mg/kg CBD, i.p. 30 min prior to PPI paradigm	30	Levin et al., 2014
MK-801 (acute, 0.3 or 0.6 mg/kg), male Sprague Dawley rats	3, 10, or 30 mg/kg CBD, i.p. injection 20 min prior to MK-801 administration. PPI paradigm started 20 min after the last injection	–	Gururajan et al., 2011
MK-801 (chronic: 1 mg/kg, 28 days), male C57BL/6J mice	30 or 60 mg/kg CBD, i.p. treatment began on 6th day of MK-801 administration. PPI paradigm was conducted on day 29	30, 60	Gomes et al., 2015a
MK-801 (acute, 1 mg/kg), male C57BL/6J mice	5 mg/kg CBD, i.p. injection 20 min prior to MK-801 administration. PPI paradigm started 5 min after the last injection	5	Long et al., 2006
Male *Nrg1* TM HET mice	1, 50, or 100 mg/kg CBD, i.p. over 21 days. PPI paradigm was done 30–45 min after the first injection and on day 21	100 (acute)	Long et al., 2012
Amphetamine (acute, 10 mg/kg) male Swiss mice	15, 30, or 60 mg/kg CBD, i.p. 30 min prior to amphetamine injection. PPI paradigm started 30 min after the last injection	15, 30, 60	Pedrazzi et al., 2015

The effects of cannabidiol on startle amplitude and PPI of healthy rodents seem to be not only dose- but also strain- and species-dependent. In male Swiss mice, cannabidiol (15, 30, or 60 mg/kg) did not affect PPI or startle amplitude (Pedrazzi et al., 2015). On the other hand, Long et al. (2006) reported that C57BL/6JArc mice acutely treated with cannabidiol (1 and 15, but not 5 mg/kg) showed increased startle amplitudes while PPI remained unaffected. In contrast, in wild type-like littermates of *Nrg1* TM HET mice, acute treatment with low concentrations of cannabidiol (1 and 50 mg/kg) as well as chronic cannabidiol treatment (1, 50, 100 mg/kg; 21 days) had no effect on startle amplitude and PPI, but acute administration of a high cannabidiol dosage (100 mg/kg) resulted in an increased startle amplitude (Long et al., 2012).

Such heterogeneous results were also observed in healthy rats. In Sprague Dawley rats, cannabidiol reduced startle amplitude (3 and 10 but not 30 mg/kg) and PPI (10 mg/kg only) in a dose-dependent manner (Gururajan et al., 2011), whereas the startle amplitude of Wistar rats was not influenced by acute cannabidiol (15, 30, 60 mg/kg) treatment, while higher dosages of cannabidiol (30, 60 mg/kg) seemed to increase PPI (Levin et al., 2014).

Interestingly, animals with transmembrane *Neuregulin 1* mutation, representing a genetic glutamatergic schizophrenia model, showed similar startle amplitude and PPI compared to their wild type like littermates. However, acute administration of 100 mg/kg cannabidiol increased not only the startle amplitude as observed in wild type like littermates, but also PPI. On the other hand, chronic cannabidiol treatment (1, 50, 100 mg/kg; 21 days) had no effect on startle amplitude and PPI in *Nrg1* TM HET or their wild type like littermates (Long et al., 2012).

Pharmacological studies mimicking glutamatergic deficits of schizophrenia revealed that pretreatment with cannabidiol (5 mg/kg) reversed PPI disruption in C57BL/6JArc mice (Long et al., 2006). In addition, chronic cannabidiol treatment (30 or 60 mg/kg) attenuated PPI impairments induced by chronic MK-801 administration in C57BL/6J mice (Gomes et al., 2015a). While cannabidiol seems to be efficacious in treating PPI impairments in mice, pretreatment with cannabidiol had no effect on PPI deficits induced by acute MK-801-injection in Sprague Dawley rats (Gururajan et al., 2011).

However, acute cannabidiol treatment also reversed PPI deficits in two other animal models of aspects of schizophrenia. First, cannabidiol (30 mg/kg) reversed PPI deficit of SHR rats but had no effects on their reduced startle amplitude (Levin et al., 2014). Second, cannabidiol (15, 30, or 60 mg/kg) attenuated the amphetamine-disruptive effects on PPI in male Swiss mice (Pedrazzi et al., 2015). Interestingly, the inhibition of anandamide hydrolysis by URB597 [selective fatty acid amide hydrolase (FAAH) inhibitor] had the same effect. Hence, the authors suggested that an increase of anandamide availability might be involved in the beneficial effects of cannabidiol.

In a nutshell, various studies showed that cannabidiol partially affected startle amplitude and PPI in healthy animals, while it had no effects in rats treated with MK-801, but reversed the PPI disruptive effects of MK-801 and amphetamine in mice as well as the PPI deficit of SHR rats.

These discrepancies, observed in both healthy wild type animals and animal models for aspects of schizophrenia, might be related to the different species/strain or experimental conditions applied. Therefore, more studies clarifying the potential antipsychotic effect of cannabidiol with regard to this specific behavioral deficit are desirable.

### Effect of Cannabidiol on Working Memory

Various paradigms are available to test cognitive performance in animals. Two studies investigating the effects of cannabidiol on cognitive performance in Δ^9^-THC- or MK-801-treated animals, respectively, used paradigms based on object and/or spatial recognition. In addition, two further studies investigated the effects of cannabidiol-rich cannabis extracts in a spatial recognition task (**Table 3**).

**Table 3:** Animal studies evaluating the effects of cannabidiol (CBD) on working memory.

Animal model	Treatment regimen and test procedure	Effective dose [mg/kg]	Reference
MK-801 (chronic: 1 mg/kg, 28 days), male C57BL/6J mice	30 or 60 mg/kg CBD, i.p. injection 30 min prior to novel object recognition test	30, 60	Gomes et al., 2015b
Δ^9^-THC (0.2, 0.5 mg/kg i.m.), male adults rhesus monkeys	0.5 mg/kg CBD, i.m. concurrently with Δ^9^-THC administration. Visuospatial Paired Associates Learning task and Self-Ordered Spatial Search started 30 min after the injections	0.5 (task selective)	Wright et al., 2013
Δ^9^-THC-rich and CBD-rich cannabis extracts, male, adult Lister rats	CBD-rich cannabis extracts (0.5, 5, 10, or 50 mg/kg CBD and up to 4 mg/kg Δ^9^-THC), i.p. 30 min prior to Delayed Matching to Sample task. In addition, CBD-rich cannabis extracts were simultaneously injected with Δ^9^-THC-rich cannabis extract injection	50 (as it contained nearly 4 mg/kg Δ^9^-THC	Fadda et al., 2004
MK-801 (0.1 mg/kg, acute), male, adult Lister rats	CBD-rich cannabis extracts (5 or 10 mg/kg CBD), i.p. concurrently with MK-801 injection, 30 min prior to Delayed Matching to Sample task. In addition, CBD-rich cannabis extracts were simultaneously injected with Δ^9^-THC-rich cannabis extract injection	–	Fadda et al., 2006

The visuospatial Paired Associates Learning task (vsPAL) and the Self-Ordered Spatial Search (SOSS) task belong to the category of spatial recognition tasks that are frequently used in non-human primates. In rhesus monkeys, acute intramuscular Δ^9^-THC administration (0.2 and 0.5 mg/kg) impaired overall trial completion accuracy and percent completed trials in the vsPAL with increasing trial-difficulty as well as trial completion accuracy in the SOSS task (Wright et al., 2013). Cannabidiol itself had no effect on cognitive performance. Interestingly, co-treatment with cannabidiol reversed the effects of Δ^9^-THC on vsPAL, but did not affect Δ^9^-THC-induced SOSS deficits.

These results are in line with a study assessing the effects of cannabidiol on novel object recognition (NOR) impairments in mice. The NOR task is often used in rodents and partially resembles vsPAL. While in vsPAL a familiar object and its former position has to be identified, NOR evaluates whether the animal recognizes a new unfamiliar object, as rodents tend to explore new objects more intensively than familiar ones. Cannabidiol (30 or 60 mg/kg) significantly reversed the NOR performance impairments observed in male C57BL/6J mice chronically treated with MK-801, but had no effect *per se* (Gomes et al., 2015b).

Furthermore, Fadda et al. (2004, 2006) investigated the effects of cannabidiol-rich cannabis extracts in a water maze based delayed-matching-to-position task (DMTP). In the first trial, animals were placed onto a platform. In the second trial animals were released into the water and had to find the platform again. Cannabidiol-rich cannabis extracts did not affect the spatial working memory of rats, although these extracts also contained Δ^9^-THC. In particular the highest dose of 50 mg/kg cannabidiol-rich extracts contained nearly 4 mg/kg Δ^9^-THC, a dose that was sufficient to impair the working memory when given alone. Thus, the authors concluded that cannabidiol is able to antagonize the cognitive impairment. However, the cannabidiol-rich extracts did not reverse memory deficits when administered concurrently with the Δ^9^-THC-rich cannabis extracts. Therefore, it has been suggested, that the cannabidiol/Δ^9^-THC ratio was not high enough to be effective (Fadda et al., 2004).

Interestingly, cannabidiol-rich extracts (5 and 10 mg/kg) were also unable to reverse working memory deficits induced by MK-801 in rats (Fadda et al., 2006). It might be that the cannabidiol dosage had simply been too low, as higher concentrations (30 or 60 mg/kg) had been shown to be effective in mice (Gomes et al., 2015b). However, even higher dosages of cannabidiol may not reverse working memory deficits induced by MK-801 in rats due to interspecies differences.

Overall, the limited data available seem to suggest that cannabidiol has potential in ameliorating not only negative symptoms but also cognitive functions. However, as task-selective differences were observed, its effectiveness might be restricted to certain aspects of cognitive functions. Therefore, further studies analyzing the effects of cannabidiol on various cognitive aspects are called for.

## Antipsychotic Potential Of Cannabidiol: Evidence From Clinical Studies

The results of the first individual treatment attempt with cannabidiol were reported in Zuardi et al. (1995). Daily administration of up to 1500 mg/day over 4 weeks resulted in decreased scores on the Brief Psychiatric Rating Scale (BPRS) and Interactive Observation Scale for Psychiatric Inpatients (IOSPI), indicating an overall improvement of psychotic symptoms. Interestingly, the clinical improvement was not increased by additional treatment with haloperidol, a first generation antipsychotic. Nearly 10 years later, Zuardi et al. (2006) published a small case series of three patients. They were treated with increasing doses of cannabidiol (maximum 1280 mg/kg) over 30 days. Importantly, one of these patients showed a slight improvement of both positive and negative symptoms. Interestingly, no side effects were reported in patients treated with cannabidiol. The first controlled, randomized, double-blind clinical trial was conducted by Leweke et al. (2012). During this 4-week trial 42 schizophrenic patients received either cannabidiol (600–800 mg/day) or amisulpride (600–800 mg/day) – a highly effective second generation antipsychotic, selectively antagonizing D_2/3_ receptors (Leucht et al., 2002). Both drugs resulted in significant clinical improvement of both positive and negative symptoms of psychosis. The efficacy of cannabidiol was comparable to that of amisulpride, but, importantly, cannabidiol revealed a superior side effect profile when compared to amisulpride. In particular, cannabidiol did not induce prolactin increase, weight gain, or extrapyramidal symptoms. Interestingly, in patients randomly allocated to cannabidiol treatment, the reduction of psychotic symptoms was significantly associated with an increase of anandamide levels in serum. This was exclusive for the cannabidiol treatment group and is in support for the hypothesis that cannabidiol’s antipsychotic effect is at least in part mediated via anandamide and potentially related to a block of its metabolization or uptake.

As reviewed in Leweke et al. (2016), four additional clinical trials with schizophrenic patients have been initiated so far. Although to date data have not been published in a peer reviewed process, the sponsor of one recent clinical trial investigating the antipsychotic effects of cannabidiol (GW42003, 1000 mg/day) as add-on medication in 88 patients suffering from schizophrenia or related disorders (e.g., schizoaffective or schizophrenia-like disorder) over a period of 6 weeks, announced that cannabidiol was consistently superior to placebo with regard to psychopathology while at the same time showing no relevant side-effect profile (GW Pharmaceuticals plc, 2015).

While cannabidiol seems to develop antipsychotic properties during at least 4 weeks of treatment, acute administration of cannabidiol (300 or 600 mg/kg) did not affect selective attention studied in 28 schizophrenic outpatients (regularly treated with antipsychotics) using the Stroop Color Word Test (Hallak et al., 2010). However, there also were no side effects reported.

Although, only few data on the antipsychotic potential in schizophrenic patients are currently available (summarized in **Table 4**), they consistently indicate a promising efficacy and favorable side-effect profile of cannabidiol. However, large-scale clinical trials are still needed to evaluate the long-term efficacy and safety of this putative new antipsychotic.

**Table 4:** Published clinical trials and case series evaluating the effects of cannabidiol in schizophrenic patients.

Design	Primary efficacy endpoint	Outcome	Reference
Single case report, open-label, treatment-resistant schizophrenia, up to 1500 mg/day CBD over 4 weeks	Psychotic symptoms (BPRS; IOSPI)	Improvement in a treatment-resistant patient	Zuardi et al., 1995
Open-label, case series (three patients), treatment-resistant schizophrenia, up to 1280 mg/day CBD over 30 days	Psychotic symptoms (BPRS)	One patient showed mild improvement in positive and negative symptoms	Zuardi et al., 2006
Double-blind, active controlled acute trial, single CBD (300 or 600 mg) or placebo administration	Stroop Color Word Test (SCWT)	No beneficial effects of single CBD administration on cognitive performance of schizophrenic patients	Hallak et al., 2010
Double-blind, active-controlled RCT with 42 acute schizophrenic patients, 600–800 mg/day over 4 weeks	Psychotic symptoms (PANSS/BPRS)	Significant clinical improvement compared to baseline on days 14 and 28 for CBD and amisulpride. Superior side-effect profile for CBD compared to amisulpride	Leweke et al., 2012

*BPRS, Brief Psychiatric Rating Scale; CBD, cannabidiol; IOSPI, Interactive Observation Scale for Psychiatric Inpatients; PANSS, Positive and Negative Syndrome Scale; RCT, randomized clinical trial.*

## Cannabidiol: Potential Mechanism Of Action

The mode of action of cannabidiol is still not fully understood, although a plethora of possible mechanisms have been proposed.

Cannabidiol and Δ^9^-THC are the most important phytocannabinoids in the cannabis plant. Therefore, it has been hypothesized that both compounds might have the same molecular target, the cannabinoid receptors. However, several binding studies showed that cannabidiol has no significant affinity at CB_1_R and CB_2_R (Devane et al., 1988; Showalter et al., 1996; Thomas et al., 1998; Bisogno et al., 2001; Jones et al., 2010). In addition, most efficacy studies found no explicit receptor response (Matsuda et al., 1990; Petitet et al., 1998; Breivogel et al., 2001; Jones et al., 2010). In fact, it has been reported that cannabidiol acts as an antagonist of CB_1_R agonists such as WIN-55212 and CP-55940 (Petitet et al., 1998; Pertwee et al., 2002; Thomas et al., 2007). As cannabidiol also inhibited internalization of CB_1_R (Laprairie et al., 2014), it has been hypothesized that the observed antagonistic activity might be based on negative allosteric modulation of CB_1_R rather than on orthosteric binding (Laprairie et al., 2015). Consistent with these findings, Laprairie et al. (2015) provided evidence that *in vitro* cannabidiol behaves as a non-competitive negative allosteric modulator of CB_1_R.

Owing to the observed significant association of the antipsychotic effect of cannabidiol with an increase of anandamide levels in serum (Leweke et al., 2012), it has been hypothesized that cannabidiol exerts its antipsychotic properties by moderately blocking FAAH, resulting in an inhibition of anandamide and of related fatty acid ethanolamide (palmitoylethanolamide and oleoylethanolamide) degradation. *In vitro*, cannabidiol inhibited FAAH in mouse neuroblastoma cell (N18TG2) membrane preparations (Bisogno et al., 2001), mouse brain microsomes (Watanabe et al., 1996), as well as homogenates of rat brain membranes (De Petrocellis et al., 2011; Leweke et al., 2012). Moreover, it has been shown that cannabidiol blocks anandamide transporters, since it inhibited anandamide uptake by rat basophilic leukemia cells (RBL-2H3) (Rakhshan et al., 2000; Bisogno et al., 2001; De Petrocellis et al., 2011). So far, one possible anandamide transporter, termed FAAH-like anandamide transporter (FLAT) has been identified (Fu et al., 2012). As FLAT seems to be a splicing variant of the *Faah-1* gene, it may be speculated that cannabidiol binds to similar binding sites of FAAH and FLAT proteins and is able to inhibit both, anandamide degradation and uptake. However, it has recently been reported that cannabidiol does not inhibit the human FAAH enzyme, but binds to fatty acid-binding proteins (FABPs) (Elmes et al., 2015), which seem to act as intracellular transporters of anandamide and other *N*-acylethanolamines (Kaczocha et al., 2009, 2012). Elmes et al. (2015) concluded that cannabidiol reduces anandamide inactivation in humans by competing with anandamide for FABPs binding. As long as FABPs are occupied by cannabidiol, anandamide cannot be transported to the FAAH enzyme, localized on the endoplasmic reticulum, resulting in elevated anandamide levels.

Although, various other molecular targets have been suggested to contribute to the antipsychotic effects of cannabidiol, their pharmacological relevance still needs to be evaluated in clinical trials.

On the one hand cannabidiol may facilitate 5-HT_1A_ receptor mediated serotonergic neurotransmission. In Chinese hamster ovary (CHO) cells transfected with the human receptor, cannabidiol displaced the 5-HT_1A_ receptor agonist [3H]8-OH-DPAT and increased [35S]GTPγS binding (Russo et al., 2005). On the other hand, cannabidiol did not displace [3H]8-OH-DPAT or stimulate [35S]GTPγS binding in rat brainstem membrane preparations, while it increased the maximal efficacy of 8-OH-DPAT at 100 nmol/L, but not at 1, 10, 31.6 nmol/L or 1 μmol/L (Rock et al., 2012). Interestingly, in mice, the anticataleptic effect of cannabidiol was prevented by the 5-HT_1A_ receptor antagonist WAY100635 (Gomes et al., 2013; Sonego et al., 2016). However, it remains unclear, whether the improvement of negative symptoms and cognitive deficits by cannabidiol involves 5-HT_1A_ receptor activation, as studies addressing this mechanism are lacking.

In addition, it has been reported that cannabidiol binds to the peroxisome proliferator-activated receptor gamma (PPARγ) *in vitro* (O’Sullivan et al., 2009; Granja et al., 2012). PPARγ regulates the expression of genes related to lipid and glucose homeostasis as well as inflammatory responses. Thus, cannabidiol may ameliorate both observed disturbances of glucose metabolism and inflammatory/immune processes in schizophrenic patients (Holmes et al., 2006; Leza et al., 2015; Rajasekaran et al., 2015) by PPARγ activation.

The activation of transient receptor potential vanilloid type 1 receptors (TRPV1Rs) has also been suggested as mechanism of action, as cannabidiol stimulated TRPV1R in HEK293-cells transiently expressing human (Bisogno et al., 2001; Ligresti et al., 2006; De Petrocellis et al., 2011) or rat TRPV1R (Iannotti et al., 2014). *In vivo*, it has been shown that pretreatment with TRPV1R antagonist capsazepine blocked the ameliorating effect of cannabidiol on MK-801 induced PPI decrease (Long et al., 2006), indicating at least a partial involvement of TRPV1R activation. However, capsazepine also blocks calcium channels (Docherty et al., 1997) and nicotinic cholinergic receptors (Liu and Simon, 1997), thus other mechanisms might contribute to this effect as well. Since TRPV1Rs also mediate the perception of spiciness, it may be expected that receptor stimulation by systemic administration of cannabidiol result in such subjective perceptions, given that this mechanism is relevant in humans at the dosage used. Yet, this side effect has not been reported or observed in clinical trials so far (e.g., Leweke et al., 2000, 2012). To date, evidence for a role of TRPV1R activation in schizophrenia is lacking, but TRPV1Rs may be indirectly involved in schizophrenia via its influence on dopaminergic (Tzavara et al., 2006) and glutamatergic neurotransmission (Fawley et al., 2014).

Furthermore, it has been suggested that cannabidiol targets GPR55 and GPR18 receptors, further subtypes of transient receptor potential receptors (TRPV2, TRPM8, TRPA1), α3 glycine receptors, adenosine receptors, μ and δ opioid receptors, nicotinic acetylcholine receptors, enzymes of the arachidonic acid cascade, ion channels like voltage-gated calcium channels or mitochondrial Na^+^/Ca^2+^ exchange, nitric oxide signaling and inflammatory cytokines (McPartland et al., 2014; Ibeas Bih et al., 2015). To date, the antipsychotic properties of cannabidiol cannot be directly linked to these possible targets. However, this does not mean that these mechanisms are not relevant for other medical conditions, e.g., epilepsy (Devinsky et al., 2014).

## Conclusion

The antipsychotic potential of cannabidiol has been investigated in various behavioral paradigms and different animal models of aspects of schizophrenia. Although the results were partially inconsistent, they indicate that cannabidiol treatment ameliorates impairments of PPI, social interaction behavior and cognition in rodents and rhesus monkeys. In addition, individual treatment attempts as well as one randomized, double-blind clinical study, demonstrated the antipsychotic potential of cannabidiol and its superior side effect profile compared to conventional antipsychotics. In addition, a recently conducted clinical trial investigating cannabidiol as an add-on medication showed promising results, although these have not yet been published in a peer reviewed process. Obviously more clinical trials are needed to substantiate the current findings, and in particular to investigate long-term efficacy and safety in larger cohorts.

However, cannabidiol seems to represent a mechanistically different and less side-effect prone antipsychotic compound for the treatment of schizophrenia, even though the underlying pharmacological mechanisms are still under debate. Nevertheless, the association between increased anandamide levels and reduced psychotic symptoms in schizophrenic patients treated with cannabidiol, points to a potentially new antipsychotic mechanism of action involving anandamide.

## Author Contributions

CR, JKM, BL, and FML performed the review of the literature and CR and FML drafted the manuscript with input from JKM and BL.

## Conflict of Interest Statement

FML is a shareholder of curantis UG (ltd.). The other authors declare that the research was conducted in the absence of any commercial or financial relationships that could be construed as a potential conflict of interest.
